# Transcriptome-Wide Identification of m^6^A Writers, Erasers and Readers and Their Expression Profiles under Various Biotic and Abiotic Stresses in *Pinus massoniana* Lamb.

**DOI:** 10.3390/ijms25147987

**Published:** 2024-07-22

**Authors:** Sheng Yao, Yidan Song, Xiang Cheng, Dengbao Wang, Qianzi Li, Jingjing Zhang, Qingyang Chen, Qiong Yu, Kongshu Ji

**Affiliations:** 1 State Key Laboratory of Tree Genetics and Breeding, Nanjing Forestry University, Nanjing 210037, China; yaosheng0817@163.com (S.Y.); songyidan126@163.com (Y.S.); chengxiang@njfu.edu.cn (X.C.); dbw@njfu.edu.cn (D.W.); liqianzi2000@163.com (Q.L.); jjzhang@njfu.edu.cn (J.Z.); 13345012802@163.com (Q.C.); 2 Key Open Laboratory of Forest Genetics and Gene Engineering of National Forestry & Grassland Administration, Nanjing 210037, China; 3 Co-Innovation Center for Sustainable Forestry in Southern China, Nanjing Forestry University, Nanjing 210037, China; 4 Beijing National Laboratory for Molecular Sciences, Peking University, Beijing 100871, China

**Keywords:** *N*^6^-methyladenosine, Masson pine, m^6^A regulators, expression profiling

## Abstract

*N*^6^-methyladenosine (m^6^A) RNA modification is the most prevalent form of RNA methylation and plays a crucial role in plant development. However, our understanding of m^6^A modification in Masson pine (*Pinus massoniana* Lamb.) remains limited. In this study, a complete analysis of m^6^A writers, erasers, and readers in Masson pine was performed, and 22 m^6^A regulatory genes were identified in total, including 7 m^6^A writers, 7 m^6^A erases, and 8 readers. Phylogenetic analysis revealed that all m^6^A regulators involved in Masson pine could be classified into three distinct groups based on their domains and motifs. The tissue expression analysis revealed that the m^6^A regulatory gene may exert a significant influence on the development of reproductive organs and leaves in Masson pine. Moreover, the results from stress and hormone expression analysis indicated that the m^6^A regulatory gene in Masson pine might be involved in drought stress response, ABA-signaling-pathway activation, as well as resistance to *Monochamus alternatus*. This study provided valuable and anticipated insights into the regulatory genes of m^6^A modification and their potential epigenetic regulatory mechanisms in Masson pine.

## 1. Introduction

Over 160 chemical modifications have been identified in RNA, predominantly within transfer RNA (tRNA) and ribosomal RNA (rRNA), where they play pivotal roles in governing RNA functionality [1]. Messenger RNA (mRNA), acting as an intermediary between DNA and proteins, also undergoes diverse chemical modifications, including *N*^7^-methylguanosine (m^7^G), *N*^6^-methyladenosine (m^6^A), 5-methylcytosine (m^5^C), *N*^1^-methyladenosine (m^1^A), pseudouridine (ψ), inosine (I), and *N*^6^, 2′-O-dimethyladenosine (m^6^Am), to fulfill its biological functions [2]. Among these modifications, m^6^A is the most prevalent internal modification observed in eukaryotic mRNA and is also present across various bacteria and RNA viruses [3]. M^6^A is a dynamic and reversible mechanism that undergoes regulation by diverse proteins, encompassing writers (methyltransferases), erasers (demethylases), and readers (m^6^A-binding proteins), which act synergistically to regulate the abundance of m^6^A [4,5,6]. Writers and erasers can bind the conserved consensus sequence RRACH (R=A or G; H=A, U, or C) to add and remove m^6^A modifications, and these modified RNAs eventually perform various functions by binding readers to the m^6^A sites [7,8,9]. The previous focus of m^6^A studies primarily revolved around animals. In recent years, with the rapid advancement of plant research, an increasing number of reports on m^6^A in plants have emerged.

In plants, m^6^A writers include MTA (homolog of human METTL3), MTB (homolog of human METTL14), FIP37 (homolog of human WTAP), VIRILIZER (VIR) (homolog of human VIRMA), and HAKAI [10,11,12,13,14]. By inhibiting the expression or knocking out of these genes, there is a resultant decrease in m^6^A levels, which subsequently affects the plant’s normal development and response to adversity. It is worth noting that disruption of MTA, FIP37 or VIR function leads to an approximate 80–90% reduction in m^6^A methylation levels [15,16,17,18]. However, it does not completely abolish m^6^A methylation. These findings suggest the involvement of unidentified writers in *Arabidopsis thaliana* for mRNA m^6^A methylation. The identification of m^6^A erasers have revealed the reversible nature of m^6^A modification and facilitated the advancement of functional studies on m^6^A. FTO [19] and ALKBH5, proteins involved in alkylated DNA repair [20], are recognized as mammalian m^6^A demethylases belonging to the Fe(II)/a-kg-dependent dioxygenase superfamily. However, no homologs of FTO have been identified in plants [21]. *A. thaliana* possesses 13 homologous proteins related to ALKBH, with 5 proteins (ALKBH9A/9B/9C/10A/10B) exhibiting similarities to ALKBH5 [22]. The demethylase activity of ALKBH9B and ALKBH10B has been confirmed in *A. thaliana*. However, LC-MS analysis does not detect any significant alteration in m^6^A content in *ALKBH9B* mutants [23]. Notably, while both FTO and ALKBH5 are localized within the nucleus, ALKBH9B and SlALKBH2 are cytoplasmic proteins, and ALKBH10B exhibits nucleo-cytoplasmic distribution. These findings suggest that plants may possess different types of erasers with distinct functions [22,24,25]. The functionality of the m^6^A is primarily dependent on m^6^A readers. The investigation of m^6^A readers in plants is currently limited and primarily focused on YTH proteins. In *Arabidopsis*, there are a total of 13 proteins containing the YTH domain, predominantly from the evolutionarily conserved C-terminal region (ECT) family [26]. The YTH domain sequences in *Arabidopsis* and animals exhibit high conservation, with continuous tryptophan residues serving as crucial sites for m^6^A binding. Therefore, these YTH-containing proteins in *Arabidopsis* possess the potential to bind m^6^A [27,28]. *ECT2* regulates the development of *Arabidopsis* epidermis by binding to m^6^A and regulating the stability of substrate mRNA [29,30]. Further experiments have demonstrated that the function of *ECT2* is redundant in comparison to that of *ECT3* and *ECT4*, suggesting that *ECT3* and *ECT4* are also m^6^A readers [31]. *CPSF30-L* is an m^6^A reader, homologous to *YTHDC1*, localized in the nucleus, and involved in selective polyadenylation (APA) regulation [32]. Additionally, recent studies have revealed an enhanced affinity of *ECT8* towards m^6^A under conditions of salt stress [33].

Masson pine, an economically valuable industrial tree in China, plays a crucial role in enhancing its economic benefits. Therefore, it is essential to study the molecular mechanisms underlying the growth, development, and stress response of Masson pine. Additionally, investigating the physiological and molecular characteristics of Masson pine as a conifer species holds significant biological value due to its distinct differences from broad-leaved trees. Although previous studies have demonstrated the close association between m^6^A modification and plant growth, development, and stress response [34], investigations on m^6^A in conifers such as Masson pine are lacking, and the identification of m^6^A-related proteins remains elusive. In this study, we utilized the transcriptome of Masson pine to identify regulators of m^6^A and comprehensively analyzed their physicochemical properties, phylogenetic relationships, and tissue expression patterns in response to hormonal and stress stimuli to predict the molecular function of these m^6^A regulators. This investigation provides a pivotal theoretical foundation for further elucidating the role and molecular mechanism of genes associated with the m^6^A regulatory pathway in coniferous species.

## 2. Results

### 2.1. Identification and Characterization Analysis of m^6^A Pathway Genes in Masson Pine

We have identified a total of seven writers, seven erasers, and eight reader proteins (a total of 22) in Masson pine. The length of transcripts, coding DNA sequence (CDS), polypeptide length, molecular weight, isoelectric point, and subcellular localization of each member of the newly identified writer, eraser, and reader families were further analyzed (Table S1). The CDS lengths of the writers range from 840 bp to 5649 bp. Therefore, *PmVIR*, with its polypeptide length of 1882 aa and molecular weight of 206.3 kDa, is the largest member of the writing family, whereas *PmMTC*, with its polypeptide length of 279 aa and molecular weight of 51.1 kDa, is the smallest member. Writers in the rice family had pI values ranging from 5.40 (*PmVIR*) to 7.10 (*PmHAKAI2*). Six of the seven writers had an acidic pI (below 7.0), whereas the *PmHAKAI2* was alkaline. Six of the seven writers were predicted to be nuclear-localized, suggesting that they contribute to the methylation of the transcriptome. Seven erasers were found to have CDS lengths ranging from 807 bp to 2001 bp, respectively. *PmALKBH7* is 666 aa in length and has a molecular weight of 72.7 kDa, making it the longest and largest eraser protein. *PmALKBH6* is the smallest eraser protein, with a molecular weight of 30.0 kDa. The pI values for *PmALKBH1*, *PmALKBH2*, *PmALKBH5* and *PmALKBH6* erasers are acidic, and the rest of the erasers are basic. Similarly, a wide range in CDS length, from 1872 bp to 2376 bp, was observed among eight reader genes. Accordingly, *PmYTHDF5* is the shortest reader protein (623 aa long, with a weight of 69.5 kDa), whereas *PmYTHDF2* is the longest (791 aa and 86.9 kDa). The subcellular localization of all m^6^A readers is predicted to be in the nucleus (Table S1).

### 2.2. Phylogenetic and Gene Characterization Analyses of m^6^A Writers, Erasers, and Readers

In order to explore the phylogenetic and evolutionary relationships among these m^6^A regulators, we generated a comprehensive phylogenetic tree utilizing 32 *A. thaliana* m^6^A regulators, 37 in *Populus trichocarpa* and 276 proteins in the nine Rosaceae species. The findings revealed that the m^6^A-associated genes could be categorized into three distinct clusters based on their phylogenetic relationships, namely the writers, readers, and erasers.

**Figure 1:**
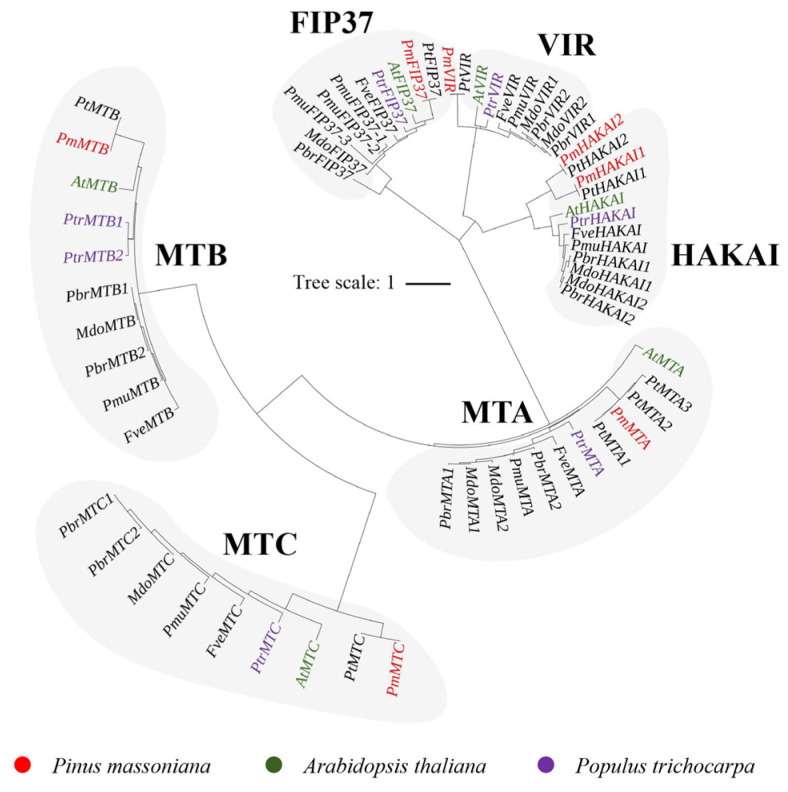
Phylogenetic analysis of m^6^A writers from *A. thaliana*, *Populus trichocarpa*, nine *Rosaceae plants* and *P. massoniana*.

**Figure 2:**
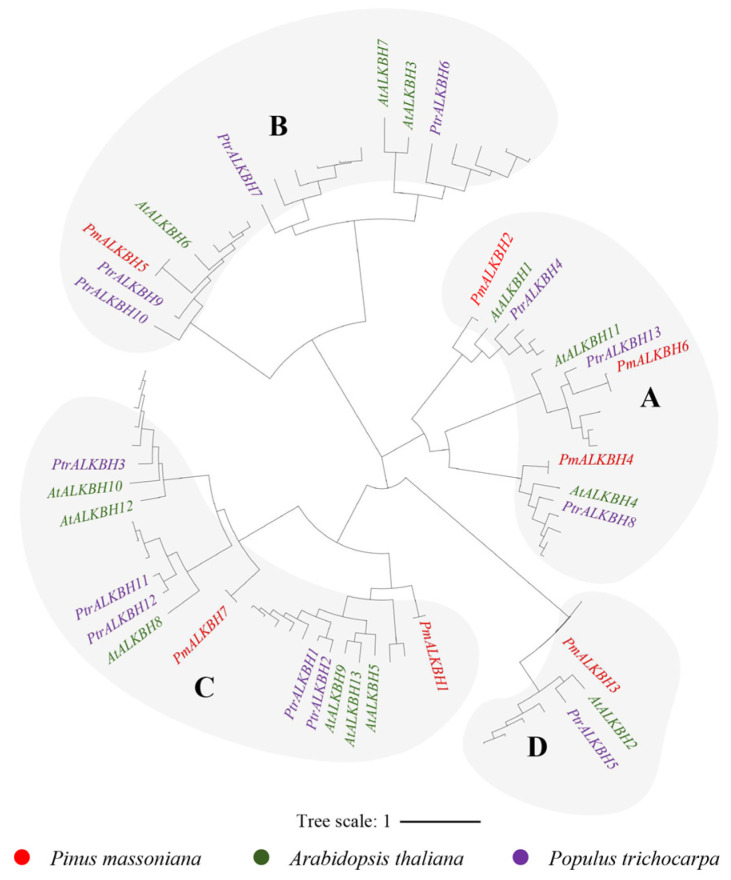
Phylogenetic analysis of ALKB family from *A. thaliana*, *P. trichocarpa*, nine *Rosaceae plants*, and *P. massoniana*. A, B, C, and D represent different subgroups.

**Figure 3:**
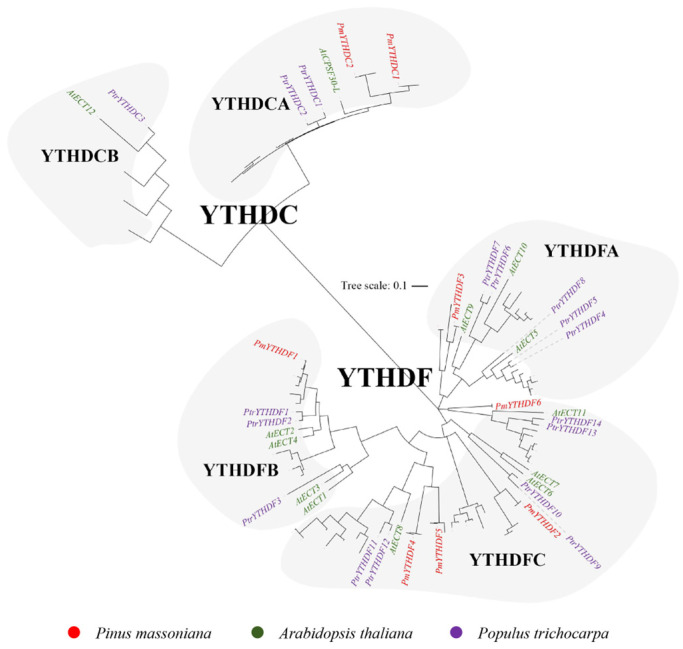
Phylogenetic analysis of YTH family from *A. thaliana*, *P. trichocarpa*, nine Rosaceae plants, and *P. massoniana*.

The readers in Masson pine can be classified into four subgroups: MT, FIP37, VIR, and HAKAI proteins, respectively. Within the MT family, there are three distinct subfamilies: MTA, MTB, and MTC (Figure 1). The eraser family in Masson pine exclusively comprises ALKBH proteins. Furthermore, the ALKBH proteins can be categorized into four branches, A, B, C, and D, and each branch includes ALKBH members of Masson pine (Figure 2). The readers in Masson pine could be further classified into two subfamilies, namely DF and DC groups. Additionally, the DF subfamily can be subdivided into three groups, DFA, DFB, and DFC, whereas the DC subfamily can be divided into two groups, DCA and DCB. It is worth noting that no member of DCB has been identified by Masson pine (Figure 3).

### 2.3. Gene Structure and Conserved Motif Analysis

**Figure 4:**
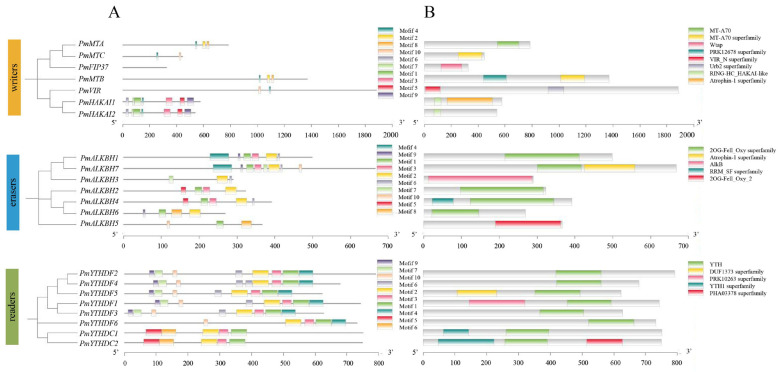
Schematics of the conserved motifs (**A**) and functional domains (**B**) of m^6^A regulators in Masson pine.

The conserved motifs and gene structures of the m^6^A regulators were identified to elucidate the sequential characteristics in Masson pine (Figure 4, Table S2). In writers, the MT-A70 domain, comprising motifs 2, 4, and 8, was distributed on the MTA. In contrast, both MTB and MTC contained the MT-A70 superfamily domain consisting of motifs 4 and 10. Furthermore, HAKAI1/2 exhibit distributed RING-HC_HAKAI-like domains formed by motifs 1 and 7. PmFIP37 featured a Wtap domain, while PmVIR contained a VIR_N superfamily domain (Figure 4A,B). In erasers, motifs 11 and 12 were observed within the 20G-Fell Oxy domain, which was ubiquitously distributed across nearly all PmALKBH members. Conversely, motif 4 exclusively manifested in group C, suggesting potential distinctive functionalities among its constituents (Figure 4A,B). In readers, the YTH domain in the PmYTHs comprised three conserved motifs (21, 22, and 23) (Figure 4A,B). Notably, the aromatic cage within the YTH domain of PmYTHs was constituted by tryptophan residues (WWW) (Figure S1).

### 2.4. Certain Authors and Readers May Participate in the Process of Phase Separation

**Figure 5:**
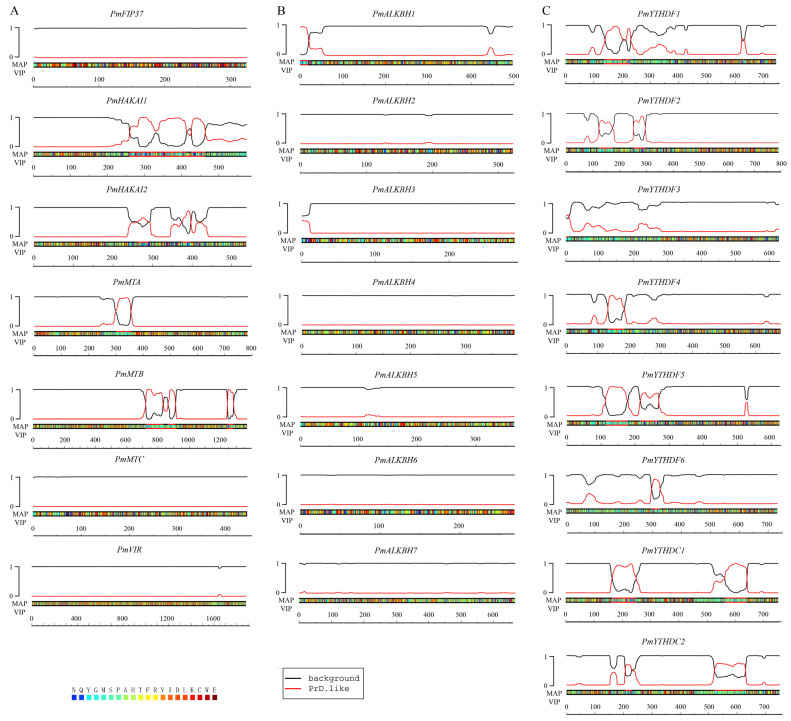
Predictions of PrLDs and disordered regions made by the PLAAC (accessed on 15 March 2024). (**A**): m^6^A writers, (**B**): m^6^A erasers, (**C**): m^6^A readers. The black line represents the background and the red line is the prediction of the prion structure region. If the red line is in the non-baseline region, it indicates that the prion structure region is at that location and the phase transition is highly likely.

Previous studies have provided evidence for the regulatory role of m^6^A site quantity and distribution in cellular mRNAs in transcriptome composition during liquid–liquid phase separation (LLPS). The analysis identified the potential participation of four writers (PmHAKAI1, PmHAKAI2, PmMTA, PmMTB) and seven readers (PmYTHDF1, PmYTHDF2, PmYTHDF4, PmYTHDF5, PmYTHDF6, PmYTHDC1, PmYTHDC2) in the LLPS process (Figure 5).

### 2.5. Expression Levels of m^6^A Regulators in Different Tissues

**Figure 6:**
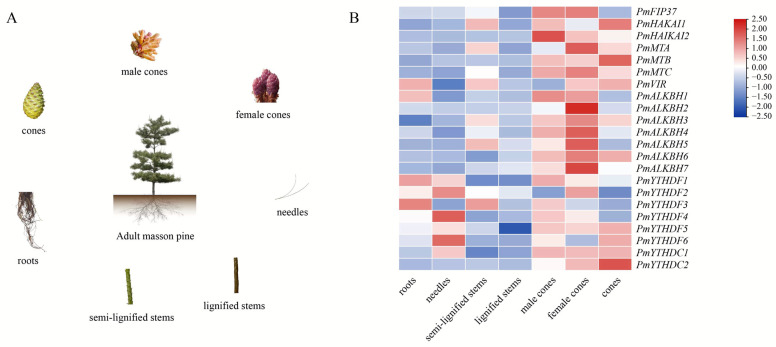
Heat map of RNA-Seq expression of m^6^A regulators in different tissues of Masson pine. (**A**): The organization diagram of Masson pine. (**B**): Heat map for tissue specific analysis.

Previous studies have demonstrated that the expression levels of key m^6^A regulators in meristems are comparatively higher than those in differentiated and mature tissues [35]. In this study, we analyzed the expression level of m^6^A regulators across various tissues of Masson pine, including roots, needles, semi-lignified stems, lignified stems, male cones, female cones, and cones (Figure 6A). Remarkably elevated expression levels of m^6^A regulators were observed in male cones, female cones, and cones, as well as in needles (Figure 6B). These findings strongly indicate a pivotal role for m^6^A in the reproductive development of Masson pine. The expression level of *PmYTHDF1* was the highest among all seven tissues (Figure S2).

### 2.6. Expression Levels of m^6^A Regulators during Conifer Development

**Figure 7:**
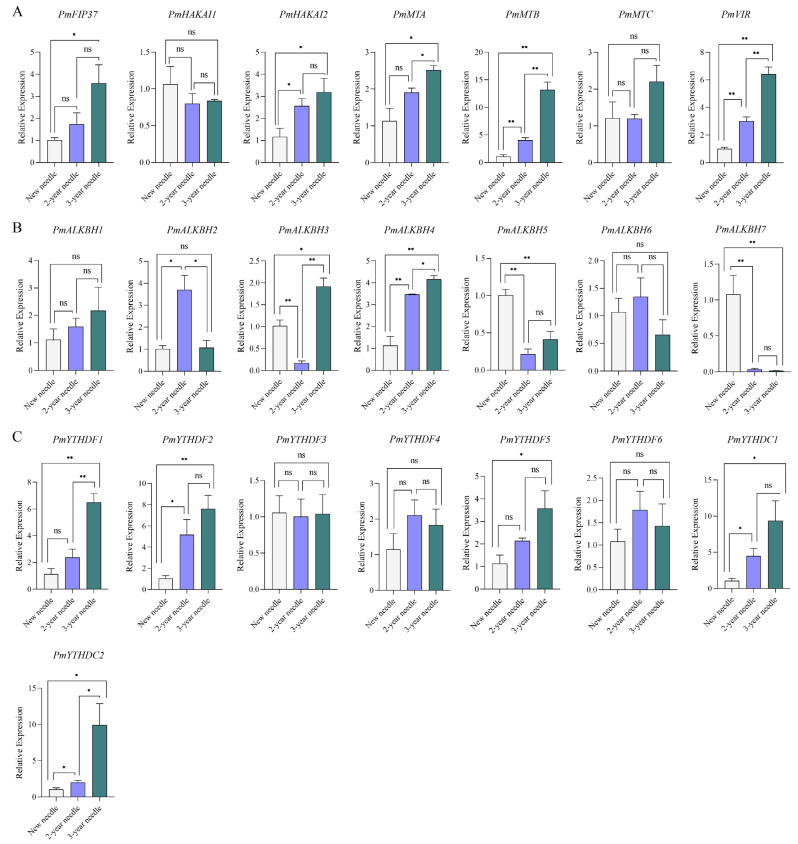
Relative expression levels of m^6^A genes during conifer development based on qRT-PCR analysis. (**A**): m^6^A writers, (**B**): m^6^A erasers, (**C**): m^6^A readers. The relative expression level was measured with the expression level of “New needle” as the control. Different numbers of “*” indicate significant differences (* *p* < 0.05, ** *p* < 0.01), ns indicate no significant difference. Data are shown as mean ± SE, with three biological replicates and three technical replicates.

Considering the distinctive characteristics of pine needles, we investigated the differential expression patterns of m^6^A regulators in needles at various developmental stages. The expression levels of PmFip37, PmHAKAI2, PmMTA, PmMTB, PmVIR, PmALKBH4, PmYTHDF1, PmYTHDF2, PmYTHDF5, PmYTHDC1, and PmYTHDC2 were generally up-regulated with increasing needle age. These genes primarily belonged to writers (5 out of 11) and readers (5 out of 11). Conversely, the expression levels of demethylated proteins such as PmALKBH5 and PmALKBH7 decreased with advancing needle age. Notably, the expression pattern of PmALKBH3 exhibited a decrease followed by an increase with increasing needle age, while that of PmALKBH2 showed an initial increase followed by a subsequent decrease (Figure 7).

### 2.7. Expression Levels of m^6^A Regulators under ABA Treatment

**Figure 8:**
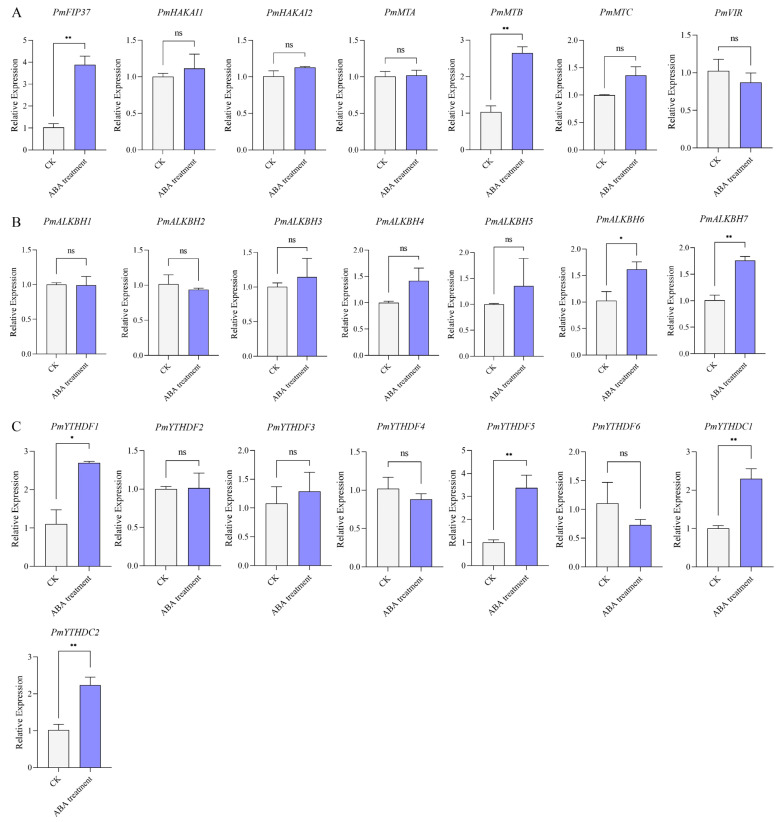
Relative expression levels of m^6^A genes under ABA treatment based on qRT-PCR analysis. (**A**): m6A writers, (**B**): m6A erasers, (**C**): m6A readers. Different numbers of “*” indicate significant differences (* *p* < 0.05, ** *p* < 0.01), ns indicate no significant difference. Data are shown as mean ± SE, with three biological replicates and three technical replicates.

Previous studies have demonstrated the involvement of m^6^A regulatory proteins in the modulation of ABA signaling through multiple pathways. Upon exposure to ABA treatment, a total of eight Masson pine m^6^A regulators, comprising two writers, two erasers, and four readers, exhibited significant alterations in their expression levels. Moreover, these genes displayed an up-regulated trend following exposure to ABA (Figure 8).

### 2.8. Expression Levels of m^6^A Regulators under Different Stresses

**Figure 9:**
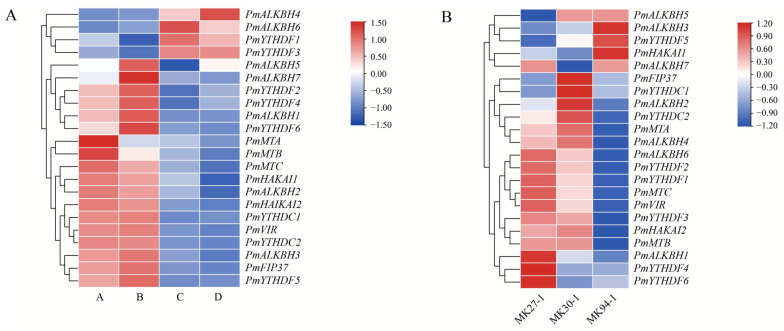
Expression pattern of m^6^A regulatory genes under biotic and abiotic stress conditions. Note: (**A**): Heat map of expression of Masson pine under drought stress. The soil moisture content for the growth of Masson pine was set to four gradients: A (80 ± 5)%, B (65 ± 5)%, C (50 ± 5)%, and D (35 ± 5)%, respectively. They were placed at 75% humidity for 60 d and subsequently sequenced. Fragments per kilobase of exon model per million mapped fragments (FPKM) values were computed to assess the expression level of m^6^A regulators. (**B**): The Masson pine clones MK27-1 and MK30-1 exhibited relatively low resistance to *Monochamus alternatus*, whereas the MK94-1 clones demonstrated comparatively high resistance against *M. alternatus*. Transcripts Per Kilobase of exonmodel per Million mapped reads (TPM) values were computed to assess the expression level of m^6^A regulators.

Numerous studies have unequivocally demonstrated the pivotal role of RNA m^6^A modification in mediating plant responses to both abiotic and biotic stresses. To comprehensively elucidate the role of m^6^A regulatory proteins in the response of Masson pine to drought stress, we employed high-throughput RNA-seq data to investigate the expression patterns of these proteins under varying degrees of drought stress (Figure 9). Remarkably, the expression levels of four key m^6^A regulators (PmALKBH4, PmALKBH6, PmYTHDF1, and PmYTHDF3) exhibited a consistent upward trend with increasing severity of drought stress. Conversely, sixteen other m^6^A regulators (PmALKBH1, PmALKBH3, PmYTHDF2, PmYTHDF4, PmYTHDF5, PmYTHDF6, PmYTHDC1, PmYTHDC2, and all writers) displayed a notable overall downward trend as drought intensity escalated. The expression levels of PmALKBH5 and PmALKBH7 exhibited a peak at drought degree B, followed by a subsequent decline with increasing severity of drought. This observed trend is noteworthy to mention; this observation strongly suggests that these particular m^6^A regulators play a crucial role in modulating the level of m^6^A modifications in plants to effectively cope with drought-induced stress (Figure 9A).

The differential expression of m^6^A regulatory proteins was analyzed between high resistance and susceptibility clones of Masson pine against Monochamus alternatus. The results revealed that the expression levels of three genes (PmALKBH3, PmYTHDF5, PmHAKAI1) were comparatively lower in the sensitive clones compared to those in the high-resistant clones, whereas the expression levels of eleven genes (PmMTA, PmMTB, PmMTC, PmHAKAI2, PmVIR, PmALKBH4, PmALKBH6, PmYTHDF1, PmYTHDF2, PmYTHDF3, PmYTHDC2) were significantly higher in the high-resistant clones. These findings suggest a potential role for m^6^A modification in influencing resistance to *M. alternatus* in Masson pine. These findings suggest that these m^6^A regulators have strong correlations with stress regulation (Figure 9B).

## 3. Discussion

N^6^-Methyladenosine, as a burgeoning research area, provides a novel perspective for biological investigations. The m^6^A modification is a reversible chemical modification. It is deposited by methyltransferases (writers), removed by demethylases (erasers), and recognized by m^6^A-binding proteins (readers) [34]. However, the m^6^A regulators have not yet been discovered in conifer species. In this study, we identified the m^6^A regulators and characterized their expression pattern in Masson pine, thus establishing a fundamental basis for further elucidating the biological function of m^6^A regulators in the growth, development, and stress response of coniferous species.

### 3.1. The m^6^A Regulators of Masson Pine May Have a Similar But Unique Molecular Mechanism to Them in Other Plants

In this study, we identified 22 m^6^A-related genes in Masson pine, including 7 writers, 7 erasers and 8 readers (Figure 1, Figure 2 and Figure 3, Table S1). The domains of these proteins exhibited a high degree of conservation (Figure 4). Phylogenetic relationships analysis showed that all of the Masson pine m^6^A writers, erasers, and readers could be divided into three groups separately. The phylogenetic analysis revealed the presence of m^6^A regulators in Masson pine across almost all subgroups (Figure 1, Figure 2 and Figure 3). It is worth noting that, similar to monocotyledonous plants, an absence of members belonging to the YTHDCB subfamily is observed in Masson pine (Figure 3). This phenomenon has also been observed in *Pinus tabuliformis* (Table S2). The findings imply that the m^6^A in Masson pine may possess a distinct molecular mechanism compared to dicotyledonous plants. However, more data are needed to support this.

### 3.2. The m^6^A Regulators May Participate in LLPS Process through PrLDs Domain

Prion-like domains can induce phase transitions, and further hardening of the droplets leads to pathological fibrous aggregation [36,37]. In mammals, prion-like domains are found in the structure of m^6^A regulatory proteins, indicating that m^6^A modified RNA is usually associated with phase separation [38,39,40,41]. This phenomenon was also found in the study of Arabidopsis m^6^A regulators. In this study, prion-like domains were found in most of the readers and writers, suggesting that these proteins have the potential to undergo phase separation (Figure 5). It is noteworthy that, among the erasers, only PmALKBH1 possesses a PrLD-like motif (Figure 5). Therefore, it can be speculated that the impact of erasers on LLPS in Masson Pine is relatively minor compared to that of writers and readers.

### 3.3. The m^6^A Regulators of Masson Pine Potentially Have a Crucial Impact on Both the Development of Reproductive Organs and the Senescence Process of Pine Needles

Understanding gene expression patterns in tissues is crucial for mining functional genes. The development of reproductive organs in plants has been demonstrated by several studies to be influenced by m^6^A regulators. AtALKBH10B regulates flowering by promoting demethylation of FT, SPL3, and SPL9 [23]. The knockout of MTA results in a reduction in m^6^A methylation, thereby impeding the transition of the developing embryo beyond the globular stage [34]. AtCPSF30-L affects the flowering of A. thaliana through the binding ability of m^6^A [32]. In our study, we found that m^6^A regulators were expressed in almost all tissues. However, the majority of m^6^A regulators displayed a predilection for expression in male cones, female cones, and cones (Figure 6). This tissue-specific expression pattern implied that m^6^A regulators in Masson pine may play a pivotal role in the reproductive growth of this species. It is worth mentioning that the expression level of PmYTHDF1 in these seven tissues was significantly higher than that of other m^6^A regulators (Figure S2), and a similar phenomenon was found in Arabidopsis [30]. This suggests that PmYTHDF1 may have a unique role in RNA modification.

Recently, multiple studies have demonstrated the involvement of ECT2/3/4 in regulating Arabidopsis leaf development [31,35]. To investigate the expression pattern of m^6^A regulators during coniferous leaf development, we examined Masson pine and found that PmYTHDF1 and PmYTHDF2, homologous genes to ECT2/3/4, exhibited significantly up-regulated expression levels during needle leaf development, suggesting their potential role in this process (Figure 7C). Furthermore, PmYTHDF5 also displayed an increasing trend throughout needle development, indicating that PmYTHDF5 is involved in needle development (Figure 7C). Notably, among all expressed genes outside of PmYTHDF1, PmYTHDF5 showed the highest level of expression, which has not been observed in studies involving other species (Table S2). Additionally, some writers (PmFIP37, PmHAKAI2, PmMTA, PmMTB, PmVIR) and erasers (PmALKBH2, PmALKBH3, PmALKBH4, PmALKBH5, PmALKBH7) exhibited differential expression patterns during needle development (Figure 7A,B). This observation warrants attention for future investigations into m^6^A regulation of needle development.

### 3.4. The m^6^A Regulators of Masson Pine Potentially Play a Pivotal Role in Drought and ABA Response and Confers Resistance Against the M. alternatus

In Arabidopsis, transcripts encoding osmotic stress response proteins tended to gain m^6^A, improving their stability under drought stress. Deficiency in ALKBH10B was associated with drought-hypersensitive phenotypes. AtALKBH10B-mediated m^6^A modification modulates the mRNA stability of several negative regulators of drought stress, including P5CS1, HVAD22D, ERD10, RD21A, RD22, CORI3, COR15B, and LOX2, by affecting alternative polyadenylation [36]. Another study demonstrated that the GhALKBH10B-mediated m^6^A modification modulates the mRNA stability of several regulators of drought stress by affecting alternative polyadenylation [37]. ECT8, acting as an ABA receptor in Arabidopsis, plays a pivotal role in the regulation of the ABA signaling pathway and response to drought stress [38]. Masson pine exhibits high tolerance to drought conditions. Surprisingly, the expression levels of all m^6^A regulators in Masson pine exhibit significant variations under different drought conditions, highlighting the potential pivotal role of m^6^A regulators in augmenting drought tolerance in Masson pine (Figure 9A).

M^6^A is also implicated in ABA signaling. For instance, AtCPSF30-L regulates the selective polyadenylation of RPN10 and FYVE1 in response to ABA [32]. ECT2/3/4 synergistically enhances target gene stability by binding PAB protein, thereby regulating the molecular mechanism of ABA response [35]. Furthermore, ALKBH10B responds to salt stress and osmotic stress and is induced by two stress-responsive hormones, ABA and JA. Similar expression patterns have been observed in tomatoes and apples [39]. ALKBH9B also modulates ABA response by controlling the mRNA m^6^A level of two negative regulators of ABA signaling, ABI1 and BES1 [40]. The m^6^A reader ECT8 is induced by ABA. Recent studies have demonstrated that ECT8 mutants exhibit hypersensitivity to ABA, highlighting the crucial role of ECT8 in abiotic stress responses [33]. Therefore, we hypothesize that m^6^A regulators in Masson pine may participate in pathways associated with ABA signaling. In our study, the expression of PmYTHDF1 (homolog of AtECT2), PmYTHDF5 (homolog of AtECT8), PmYTHDC1, PmYTHDC2 (homolog of AtCPSF30-L), and PmALKBH7 (homolog of AtALKBH10) were significantly enhanced upon induction with ABA (Figure 8). This suggests that m^6^A is relatively conserved in the ABA regulatory pathway.

It has been discovered that m^6^A plays a significant role in the interaction between plants and viruses, as well as pathogenic fungi. Recent advancements have also shed light on the involvement of m^6^A in insect resistance mechanisms. PxMETTL3 and PxMETTL14, repress the expression of JHE to induce an increased JH titer, mitigating the fitness costs associated with a robust defense against the Bt pathogen [41]. However, the enhancement of insect resistance in plants through m^6^A modification has not been documented in existing studies. In our study, we identified 14 m^6^A regulators that exhibited significantly disparate expression levels between two phenotypes of high-resistance and susceptibility clones against *M. alternatus* (Figure 9B). Nonetheless, our findings strongly imply a significant correlation between these m^6^A regulators and the resistance of Masson pine to *M. alternatus*.

In conclusion, the m^6^A regulators play a pivotal role in plant growth, development, and stress response. The identification and expression pattern analysis of m^6^A regulators in Masson pine establish a fundamental basis for further investigations into the functional roles of these genes. Furthermore, this identification provides a theoretical framework and direction for the application of coniferous tree RNA epigenetic molecular breeding.

## 4. Materials and Methods

### 4.1. Plant Materials

The seven tissue samples and coniferous specimens representing the three distinct age cohorts of Masson pine were specifically obtained from a 10-year-old tree situated within the Washan state-owned forest farm, Quanjiao, Anhui Province, China (32°10′ N, 118°27′ E). One-year-old Masson pine seedlings, obtained from the seed orchard of a Baisha state-owned forest farm, Shanghang, Fujian Province, China (25°150′ N, 116°620′ E), were used in this study. Individuals of the same clones with similar heights, uniform growth, and strong growth potential were selected as the test materials and subsequently moved into a growth chamber to recover for 15 d. The growth conditions were 10 h light/14 h dark cycles at 25 °C in the chamber. The expression patterns of m^6^A regulators were investigated following treatment with ABA. A 100 mL solution of ABA (200 mg·L*^−^*^1^) was applied as a foliar spray on the experimental seedlings every morning from 9:00 to 10:00. On the 8th day, the middle and upper needles of the seedlings were uniformly collected.

### 4.2. Identification of m^6^A Regulators: Writers, Erasers, and Readers in Masson Pine

**Figure 10:**
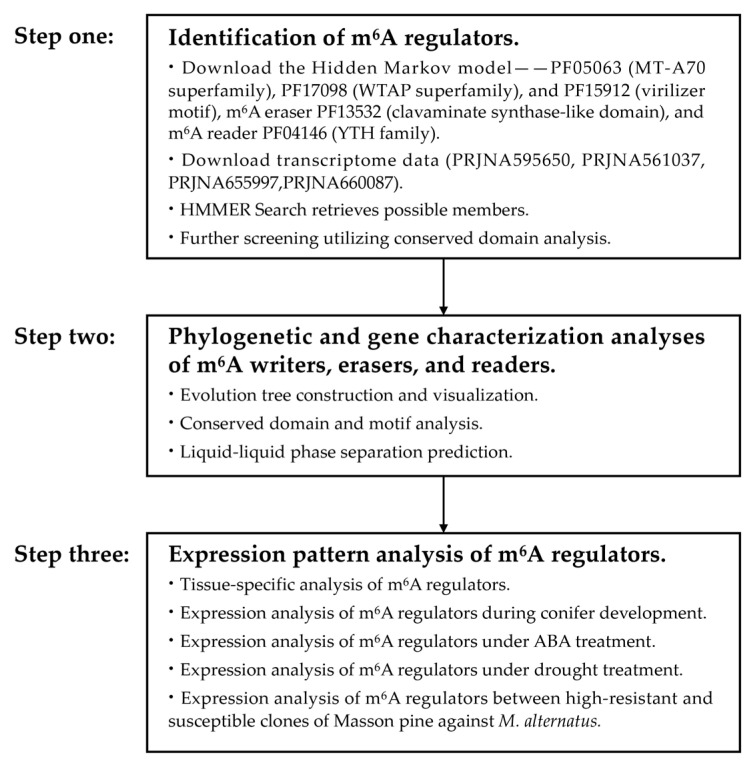
Block diagram of the research stepwise procedure.

The transcriptome data for Masson pine were obtained from the previously identified drought stress transcriptome (PRJNA595650), CO_2_ stress transcriptome (PRJNA561037), young shoots transcriptome (PRJNA655997), Masson pine inoculated with the pine wood nematode transcriptome (PRJNA660087). Hidden Markov Model (HMM) profiles of m^6^A writers PF05063 (MT-A70 superfamily), PF17098 (WTAP superfamily), PF15912 (virilizer motif), m^6^A eraser PF13532 (clavaminate synthase-like domain), and m^6^A reader PF04146 (YTH family) were employed for HMM searches in Masson pine. To ensure gene integrity, sequences with overlapping regions were excluded, while the CDD tool was utilized to verify conserved domains (Figure 10).

### 4.3. Sequence Analysis

The molecular weights and isoelectric points of the identified m^6^A regulators were determined using tools available on the ExPASy website. The molecular weights and isoelectric points of the identified m^6^A regulators were determined utilizing computational tools provided by the ExPASy website. The subcellular localization of m^6^A regulators was predicted and analyzed using PSORT (accessed on 14 March 2024). Previously published articles were used to identify m^6^A regulators of *A. Thaliana* and 9 Roseaceae plants [42], while the sequence data for *P. trichocarpa* m^6^A regulators were downloaded from NCBI. Multiple sequence alignments of the MT-A70 superfamily, ALKB family, and YTH family in nine Rosaceae species and Arabidopsis were performed using MUSCLE v5. The resultant comparative dataset was employed to generate maximum likelihood phylogenetic trees comprising 1000 bootstrap replicates via IQ-TREE (accessed on 14 March 2024).

### 4.4. Conserved Motifs and Functional Domains Analyses

The conserved motifs of m^6^A regulators were analyzed using Multiple Expectation Maximization for Motif Elicitation (MEME), with the parameters being set to a minimum and maximum motif width of 6 and 50, respectively. Additionally, a maximum number of motifs at 10 was considered. Functional domain annotation was performed utilizing the NCBI CDD (Figure 10).

### 4.5. LLPS Prediction

The PrLDs and disordered regions are predicted using the PLAAC (accessed on 15 March 2024). The visualization of the predicted outcomes is facilitated by an AI-based approach.

### 4.6. RNA Extraction and Quantitative Real-Time Reverse Transcription PCR

The total RNA was extracted using the RNAprep Pure Kit (DP441, Tiangen Biotech, Beijing, China). The concentration and purity of RNA were measured using a NanoDrop 2000 spectrophotometer (Thermo Fisher Scientific, Waltham, MA, USA), while the integrity of RNA was assessed through 1.2% agarose gel electrophoresis [43]. First-strand cDNA synthesis was performed using the One-step gDNA Removal and cDNA Synthesis Kit (AT311, TransGen Biotech, Beijing, China). Primers for quantitative real-time reverse transcription PCR (qRT-PCR) were designed with Primer 5.0 software (Table S3). SYBR Green reagents were utilized for target sequence detection. Each PCR mixture (10 µL) consisted of 1 µL of diluted cDNA (20× dilution), 5 µL of SYBR Green Real-time PCR Master Mix, 0.4 µL of each primer (10 µM), and 3.2 µL of ddH_2_O. The PCR program comprised six stages: preincubation at 95 °C for 60 s; amplification with denaturation at 95 °C for 15 s, annealing at 60 °C for 15 s and extension at 72 °C for 10s repeated 40 times; melting curve analysis with denaturation at 95 °C for 0.5 s; and annealing at 60 °C for 1 min. The quality of PCR products was evaluated based on melting curves. TUA (α-tubulin) served as an internal control. Three independent biological replicates with three technical replicates per biological replicate were examined. Quantification was achieved by comparing cycle threshold (Ct) values, and gene expression levels were calculated using the 2^−∆∆Ct^ method. Significance was determined by *t*-test analysis conducted in SPSS 26.0 (IBM, New York, NY, USA) (* *p* < 0.05,** *p* < 0.01).

### 4.7. RNA-Seq Data Analysis of m^6^A Regulators

The RNA-seq data of Masson pine under drought treatment were acquired from the NCBI database (PRJNA595650). The RNA-seq data comprised high-resistant and susceptible clones of Masson pine against *M. alternatus* transcriptome (Table S4) in our group. Fragments per kilobase of the exon model per million reads mapped (FPKM) values were calculated to estimate the abundance of m^6^A transcripts. TBtools (Toolbox for Biologists) software (v2.034) was used to create heat maps of partial genes based on the values of log_2_(FPKM+1), and analyses were performed at the row scale.

### 4.8. Statistical Analysis

The statistical analysis was performed using GraphPad Prism v8.0.2 software. One-way ANOVA was employed to compare mean differences, and statistical significance was considered at * *p* < 0.05, ** *p* < 0.01 levels. Untreated samples were utilized as a control for significant gene expression analysis.

## 5. Conclusions

In this study, we initially identified 22 m^6^A-related genes in Masson pine, including 7 writers, 7 erasers, and 8 readers. The domains of these proteins exhibited a high degree of conservation. PrLDs were found in the readers and writers, suggesting that these genes may be involved in the process of liquid–liquid phase separation. The tissue expression analysis suggests that the m^6^A-related genes of Masson pine may potentially contribute to the development of reproductive organs and the senescence process of needles. The expression analysis of stress treatment revealed the sensitivity of m^6^A-related genes to ABA and drought treatments. Moreover, we found that m^6^A-related genes exhibited significantly disparate expression levels between two phenotypes of high-resistance and susceptibility clones against *M. alternatus*, suggesting that m^6^A may confer resistance against the *M. alternatus*. These findings provide a foundation for future functional analysis of m^6^A-related genes.

## Data Availability

Data are contained within the article and supplementary materials.

## Supplementary Materials

The supporting information can be downloaded at: https://www.mdpi.com/article/10.3390/ijms25147987/s1.
