# Supplementary material for: Transcriptome-Wide Identification of m6A Writers, Erasers and Readers and Their Expression Profiles under Various Biotic and Abiotic Stresses in Pinus massoniana Lamb
Source: Int J Mol Sci. 2024 Jul 22;25(14):7987. doi: 10.3390/ijms25147987 (PMC11277107; doi:10.3390/ijms25147987)
Supplement: Supplementary file 1 [file ijms-25-07987-s001.zip › Supplementary Figures.pdf]

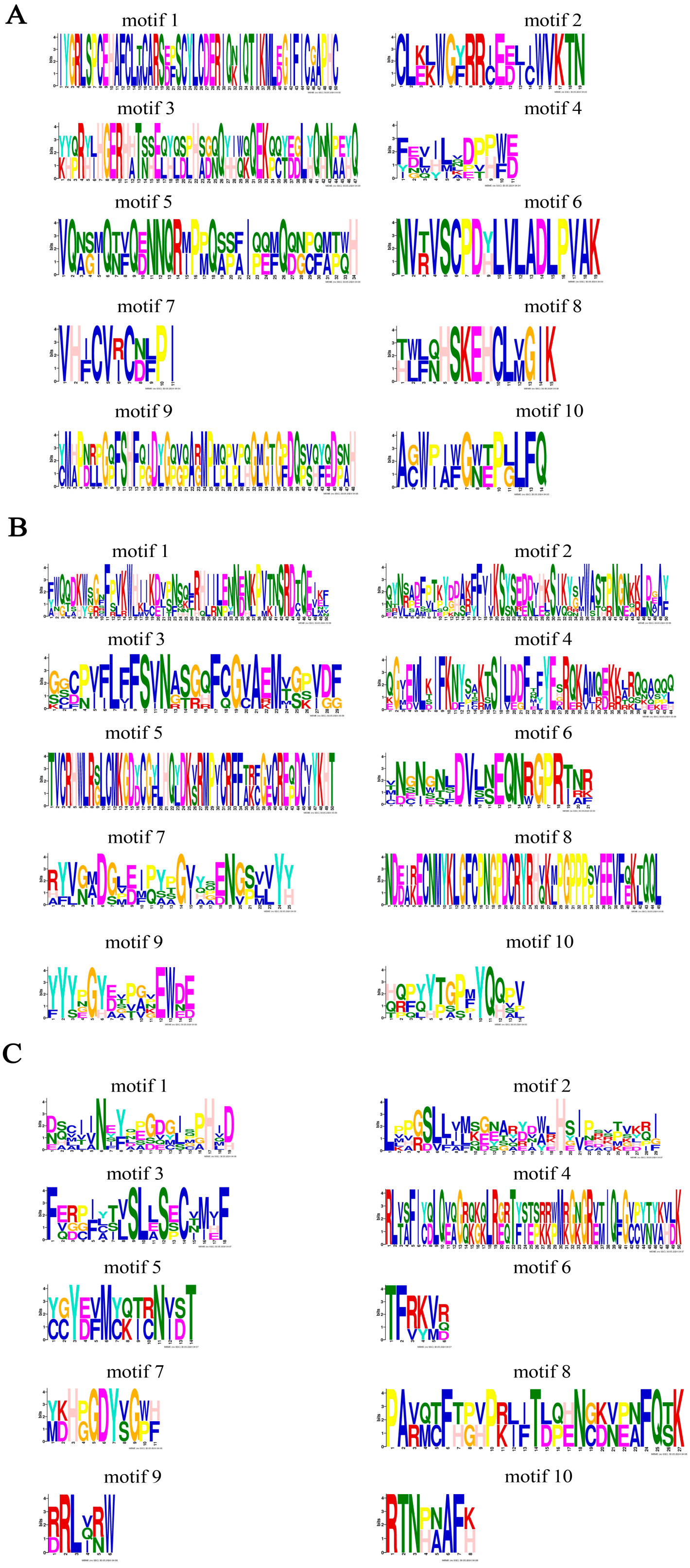

Figure S1. Sequence logos for conserved motifs identified in m^{6}A writers (A), erasers (B) and readers (C) by MEME analysis.

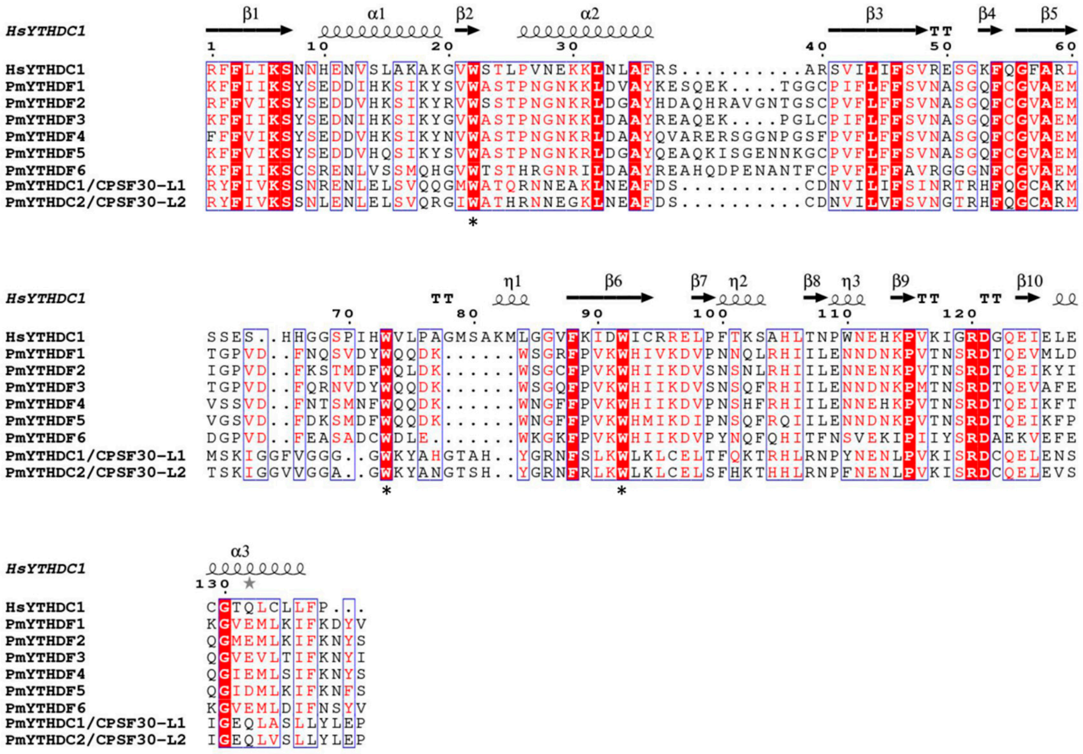

Figure S2. Sequence alignments of YTH domain in PmYTH family proteins. Asterisks indicate the tryptophan position.

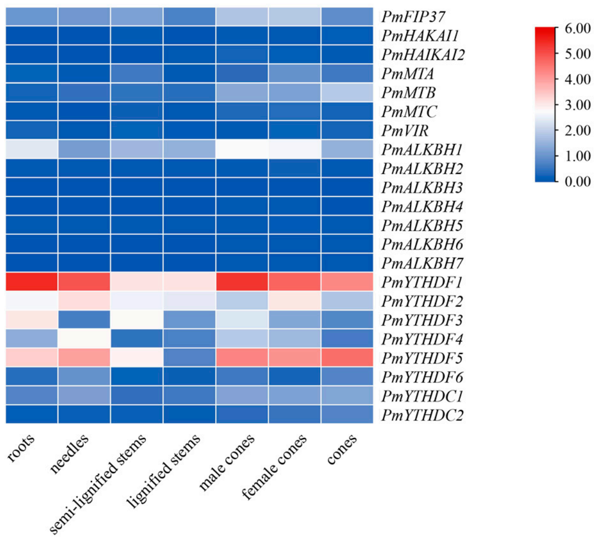

Figure S3. The expression level of PmYTHDF1 was the highest among all seven tissues.
